# Serial Changes in Intestinal Stenotic Stiffness in Patients with Crohn’s Disease Treated with Biologics: A Pilot Study Using Ultrasound Shear Wave Elastography

**DOI:** 10.5152/tjg.2023.22768

**Published:** 2023-10-01

**Authors:** Hiroshi Matsumoto, Jiro Hata, Hiroshi Imuamura, Shogen Yo, Momoyo Sasahira, Hiraku Misawa, Motoyasu Oosawa, Osamu Handa, Eiji Umegami, Akiko Shiotani

**Affiliations:** 1 Division of Internal Medicine, Department of Gastroenterology, Kawasaki Medical School, Matsushima, Kurashiki, Okayama, Japan; 2 Department of Clinical Pathology and Laboratory Medicine, Kawasaki Medical School, Matsushima, Kurashiki, Okayama, Japan

**Keywords:** Ultrasound, shear wave elastography, stricture, tissue stiffness, Crohn’s disease

## Abstract

**Background/Aims:**

Intestinal strictures represent an important serious complication of Crohn’s disease. Shear wave elastography is a promising noninvasive ultrasound technique for assessing tissue stiffness. This study aimed to evaluate stiffness in the areas of intestinal stricture in patients with Crohn’s disease using shear wave elastography and the changes in stiffness after biologics.

**Materials and Methods:**

We enrolled 21 Crohn’s disease patients having intestinal stricture. The patients consisted of 3 groups, which were the infliximab naïve (n = 6) group, the ustekinumab naïve (n = 8), and the bio-switch from infliximab to ustekinumab (n = 7) group. Bowell wall thickness was examined by ultrasound sonography, and the stiffness of Crohn’s disease stricture lesions was evaluated using Shear wave speed before and 1 year after anti-tumor necrosis factor-alpha antibody infliximab, anti-interleukin 12/23 antibody ustekinumab, and bio-switch from infliximab to ustekinumab.

**Results:**

Bowell wall thickness was significantly improved after infliximab, ustekinumab, and the bio-switch. However, shear wave speed indices only in the ustekinumab group significantly decreased after treatment (*P* = .028), but not in the other group.

**Conclusions:**

Shear wave elastography might be a useful method to evaluate stiffness in the areas of intestinal stricture in patients with Crohn’s disease treated with biologics. However, a prospective randomized study evaluating the development of obstruction after biological treatment is needed to validate the study findings.

**Main Points**

- Intestinal stricture is an important serious complication of Crohn’s disease.
- We evaluated stiffness in the areas of intestinal stricture in patients with Crohn’s disease. using share wave elastography and the change in stiffness after infliximab and ustekinumab.
- Shear wave speed was significantly decreased only after ustekinumab.
- Shear wave elastography might be a useful method to evaluate stiffness in intestinal stricture in patients with Crohn’s disease.

## Introduction

Crohn’s disease (CD) is a transmural disease in which progressive inflammation leads to intestinal wall thickening and fibrous processes. The use of biologics for the treatment of CD has been increasing; however, treatment is challenging due to the development of new therapies with different mechanisms of action. Tumor necrosis factor-alpha (TNF-α) antagonists, such as infliximab (IFX), adalimumab, and certolizumab, have revolutionized the treatment of moderate-to-severe CD patients with an inadequate response to standard medication.^1^ Although IFX treatment is effective in patients with CD, it causes strictures in some patients.^2^ On the other hand, treatment options for CD have increased due to the development of new biologics such as ustekinumab (UST), an anti-p-40, and vedolizumab, an anti-α4β7 integrin.^3^

A recent study has reported the comparative efficacy of IFX and UST in patients with CD, indicating similar rates of week 6 clinical remission and response.^4^ However, it has been hypothesized that IFX and UST may have different effects on healing because UST may lead to suppression of fibrogenesis and formation of stenosis. Ustekinumab suppresses the cytokine pathway of Th1 and Th17 cells by suppressing both interleukin (IL)-23 and IL-12 production.^5^ In addition, UST may decrease the production of transforming growth factor (TGF)-β by Th1 cells via macrophages and that of IL-22 and IL-17 by Th17 cells, suppressing the proliferation or transformation and function of myofibroblasts.

Stricturing CD is a significant clinical problem developing obstruction and ileus. Fibrostenotic disease results from not only an initial trigger of untreated inflammation but also overstimulated repair mechanisms, causing excess fibrosis.^6^ CD-associated stricture is characterized by the deposition of extracellular matrix proteins such as fibronectin and collagen. According to population-based studies using the Montreal classification, the probability of progression to stricturing CD is approximately 15% and 21.6% at 10 and 20 years, respectively.^7^ Therefore, it is important to analyze the types of intestinal stricture, degree of stenosis, and stiffness to select treatments against the intestinal stricture, such as anti-inflammatory drugs, endoscopic balloon dilation, and surgical resection.

Intestinal strictures are clinically evaluated mainly by cross-sectional images such as intestinal ultrasound (IUS), computed tomography (CT), and magnetic resonance imaging (MRI) examinations because they allow sophisticated assessment of the entire intestinal wall.^8^ In particular, IUS is a noninvasive, non-radiation, broadly available, and accurate diagnostic tool to evaluate disease activity and complications, monitor disease progression, and assess therapeutic response in patients with CD.^9,10^ Point-of-care bowel ultrasound (US) has a sensitivity of 80%-90% and a specificity of 94%-98% in properly discriminating inflammatory from noninflammatory diseases in patients with abdominal symptoms and leads the modification of treatment strategies in more than 60% of CD patients.^11,12^ The treat-to-target strategy for inflammatory bowel disease shifts the goal of treatment to long-term prevention of complications and proposes close monitoring of disease activity.^13^

Ultrasound elastography is a promising, noninvasive technique for assessing tissue stiffness. There are 2 major types of elastography shear wave elastography (SWE) and strain elastography (SE). In SE, the strain ratio is correlated with the severity of bowel fibrosis.^14^ In the SWE method, an initial US push pulse that induces shear waves perpendicular to the US beam is applied to the tissue. Shear wave elastography measures the scissoring speed of a shear wave induced by an acoustic radiation force impulse, while SE assessment is a derivative of comparison between targeted and surrounding tissues after an external pressure induced by an operator presenting as a color-coded elastogram. The interpretation of SE is more subjective than that of SWE due to the diagnostic method itself, and SWE is more advantageous than SE as the measurements are objective and do not depend on the surrounding tissues. Thus, SWE is more reliable and reproducible than SE.^15^ Shear wave elastography has also been shown to be useful for differentiating benign lesions from malignant lesions in the breast, prostate, and thyroid.^16-18^ Recently, some reports have demonstrated that US elastography is a useful examination tool to analyze bowel wall thickness (BWT) and stricture in patients with CD.^19,20^ Ding et al^21^ evaluated the diagnostic performance of both SE and SWE for intestinal stenosis in patients with CD, indicating the better performance of SWE in evaluating and differentiating intestinal stenosis in CD. However, no studies have evaluated the impact of biologics on tissue stiffness in patients with CD stricture lesions using SWE. 

Therefore, this study aimed to evaluate and compare the change of stiffness of CD stricture lesions using SWE after anti-TNF-α antibody IFX, anti-IL 12/23 antibody UST, and bio-switch from IFX to UST. 

## Materials and Methods

### Patients and Examination

Outpatients and inpatients with stricturing CD scheduled to undergo IUS were enrolled in this study from December 2019 to January 2021. Patients who underwent SWE before and 1 year after IFX and UST treatment and bio-switch from IFX to UST were selected. Patients treated with other biologics were excluded due to the small sample size. Patients lacking SWE images, either before or after treatment were also excluded.

**Intestinal Ultrasound**

Intestinal ultrasound was performed using US system Aplio i 900 (Cannon Medical system, Japan) with a 7 MHz linear transducer (3-11 MHz, PLI-705BX) in the most affected bowel segment, mainly ileocecal lesion in CD. All patients underwent IUS in the supine position after at least 6 hours of fasting. These procedures were performed by a single gastroenterologist (J.H.), with >35 years of experience in IUS examination. The disease site was defined as pathological wall thickness based on BWT >3 mm for ileum and >4 mm for colon and ileocecal area.^10,22^

### Ileocecal/Ileal Stricture Lesions

The intestinal stricture was determined based on colonoscopy small bowel radiography or IUS findings. A stricture lesion was defined as an unpassing lesion on the colonoscopy (scope dimer 13.2 mm, CF-HQ290L, 290I, Olympus, Tokyo, Japan) or luminal diameter <3 mm with/without oral side expansion on small bowel radiography or IUS. In addition, a stricture lesion was defined as a wall thickness of >4 mm by IUS. Most BWT lesions were evaluated in patients with multiple small bowel stricture lesions. 

**Shear Wave Elastography**

**Figure 1.**
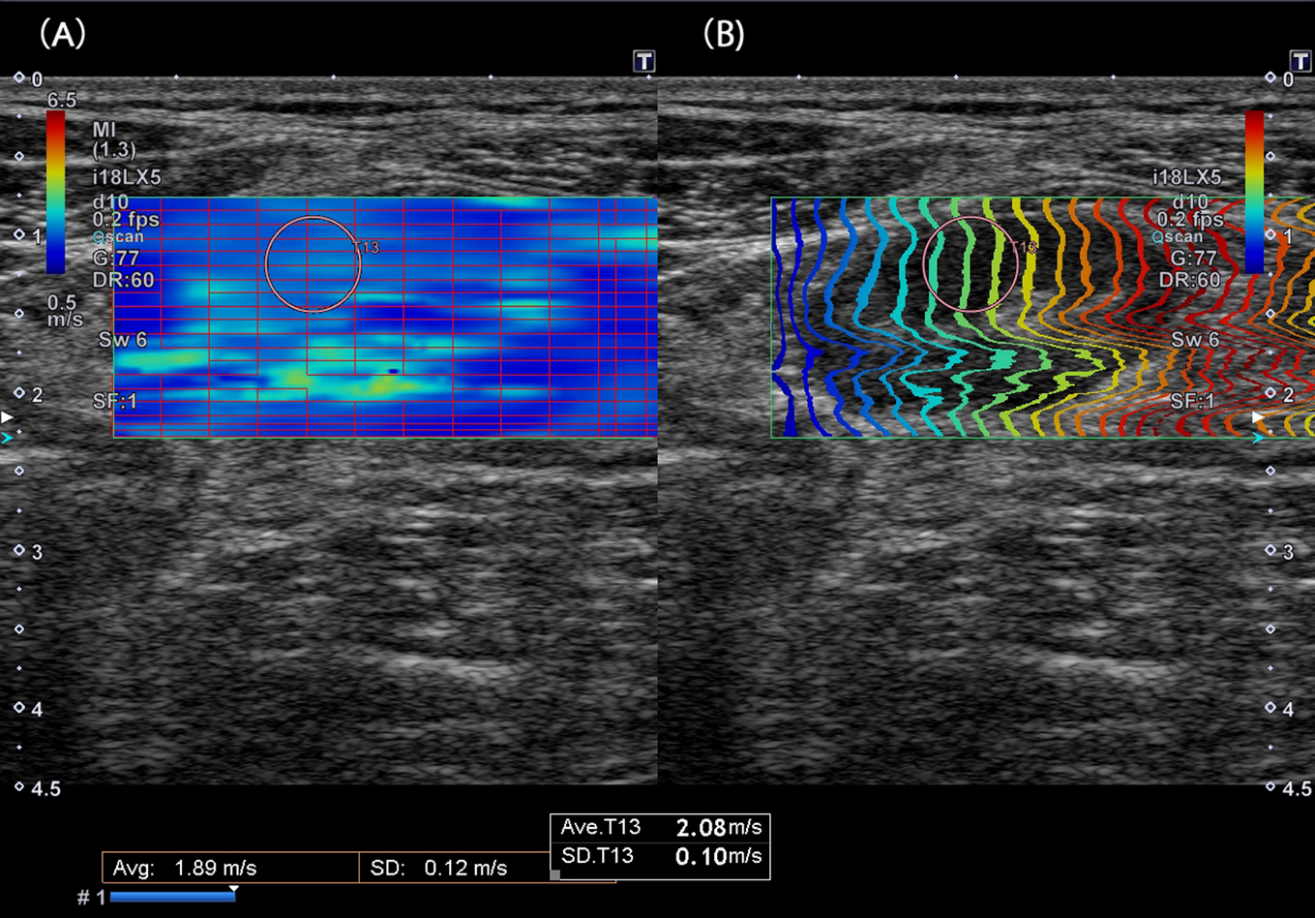
Shear wave elastography (SWE) image. (A) The rectangle has 96 small rectangles surrounded with/without a red line. The rectangle surrounded by a red line indicates that the SD is <10% of the measured mean shear wave speed (SWS), making the regions of interest (ROIs) in the rectangle reliable. On the other hand, the rectangle surrounded without a red line indicates that the SD is >10% of the measured mean SWS, making the ROIs in the rectangle without a red line unreliable. (B) Propagation map. The waveform of the measurement point is not distorted. The measured ROI (Ave T13) is 2.08 m/s, and the SD T13 is 0.10 m/s in the middle of the below table. SD T13 is lower than that of Ave T13 (2.08)/10 = 0.208. Thus, this ROI is an unsatisfied area to be measured. In addition, it can be confirmed that the ROI is in the rectangle without a red line, as seen in A, and the waveform of the ROI is not distorted, as seen in B.

Elasticity was quantified using SWE by measuring the scissoring speed induced by acoustic radiation force impulse using the same US system. During shear wave speed (SWS) measurement, the US inspector was performed without unnecessary transducer compression to prevent the increase in SWS due to technical error. With respect to the full bowel wall elasticity in stenotic lesions, at least 5 regions of interest (ROI) of the stenotic bowel segment were measured. The ROIs were placed at the position of the full thickness of the bowel wall without the surrounding tissues and luminal content. The SWS in each ROI was measured more than 5 times, and the mean value in each ROI was calculated, excluding maximum and minimum data. Data on inadequate ROIs were also excluded. An adequate ROI is defined below (Figure 1): (A) the rectangle surrounded by a red line indicated that the SD was <10% of the mean SWS value and (B) in the propagation map, the waveform of the measurement point was not distorted. 

### Ethics Committee Approval

All methods were performed in accordance with the Declaration of Helsinki and complied with relevant guidelines and regulations. Ethical approval was obtained from Kawasaki Medical School Ethics and Medical Research Committee (no. 3749). Written informed consent was achieved from each research subject before enrollment. All patients were enrolled at the Division of Gastroenterology of Kawasaki Medical School Hospital. The study was registered at the University Hospital Medical Information Network Center (UMIN 000037596). 

### Statistical Analysis

Values are presented as mean ± SD or median and interquartile range (IQR) (25%-75%) normally or nonnormally distributed data, respectively. Category data, presented as counts with percentages, were analyzed using the chi-square test. The demographic characteristics of patients and values of US parameters are expressed as the median with a range of measurements. Data were compared among 3 groups by Kruskal–Wallis analysis, chi-square test, or one-way analysis of variance test and compared between before treatment and after treatment using the paired *t*-test and Wilcoxon rank sum test. All analyses were performed using Statistical Package for the Social Sciences (SPSS) software (version 26.0)(IBM Corp.; Armonk, NY, USA). *P* <.05 were considered significant.

## Results

### Demographic Characteristics of the Patients with Crohn’s disease

Patients were divided into the 3 groups-IFX naïve (n = 6), UST naïve (n = 8), and bio-switch (n = 7) groups. The duration of CD and biologics treatment was significantly longer and operation history was significantly more frequent in the bio-switch group than in the other groups, whereas the duration of UST was not significantly different between the bio-switch group and the UST group (Table 1). 

Strictures of Bauhin’s valve were detected in 17 of 21 (80.9%) patients. The remaining patients showed ileal strictures on small bowel radiography. Ileal strictures were detected in 2 (33%) patients in the IFX group, and the strictures of Bauhin’s valve were detected in 4 (50%) patients in the UST group. The number of patients with these strictures was lower than that in the other groups; however, the frequency of strictures was not significantly different among the 3 groups (Table 1). 

The median baseline data of CD activity indices (CDAI) and C-reactive protein (CRP) levels were higher and the median baseline hemoglobin (Hb) and albumin (Alb) values were lower in the IFX group than in the other groups, although the differences were not significant. After treatment, serum CRP levels were significantly higher and Alb levels were significantly lower in the bio-switch group than in the other groups. All values of CDAI, CRP, the Hb, and the Alb improved after IFX treatment; however, the only values of CRP and Hb improved in the UST group and only the values of CDAI improved in the bio-switch group (Table 1). 

**Comparison of the Change of Bowel Wall Thickness, Shear Wave Speed Analyzed by Ultrasound, and Crohn’s Disease with Biologic Treatment**

**Figure 2.**
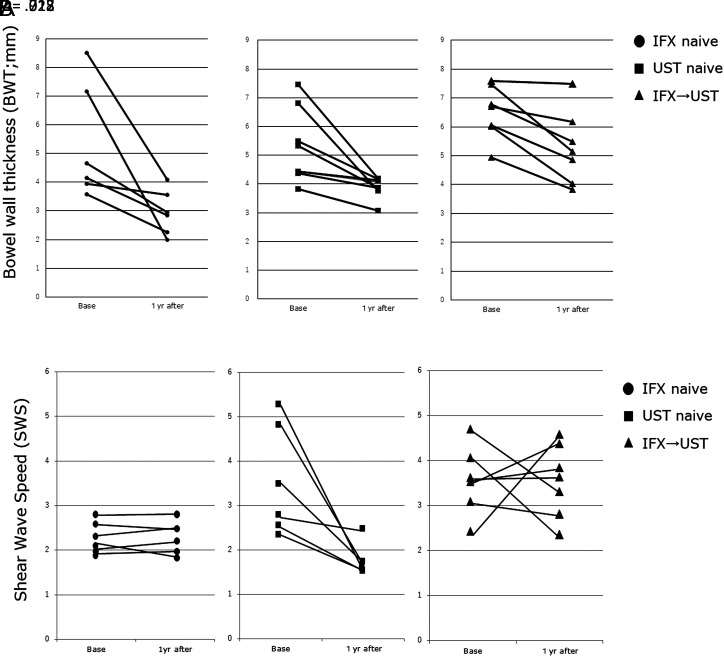
Change in the bowel wall thickness (BWT) and shear wave speed (SWS) in the 3 treatment groups—infliximab (IFX), ustekinumab (UST), and bio-switch (IFX→UST) groups. Data were compared between the 2 groups using the Wilcoxon rank sum test. (A) BWT and (B) SWS.

The median baseline BWT indices were not significantly different among the 3 groups. However, the median BWT after treatment was significantly different among the 3 groups; it was the highest in the bio-switch group (5.15 mm) and decreases in the UST group (3.97 mm) and the IFX group (2.89 mm) (Table 2). Bowel Wall Thickness significantly decreased 1 year after treatment in all 3 groups (IFX group, *r* = .028; UST group, *r* = .012; bio-switch group, *r* = .018, Figure 2A). 

In contrast, the median SWS at baseline and after treatment were significantly different among the three groups and were higher in the bio-switch group than in the other groups. The median SWS reduction was significantly lower in the UST group than in the others (UST group: −1.58 vs. IFX group: −0.22, *P = .*029; UST: −1.58 vs. bio-switch: −0.28 *P = .*013; Table 2). However, no significant differences were observed between the IFX and the bio-switch groups. Shear wave speed significantly decreased after treatment in the UST group (from 2.81 m/s to 1.65 m/s, *P = .*028) but not in the other group (IFX group: from 2.24 m/s to 2.07 m/s, *P = .*715; bio-switch group: from 3.5 m/s to 3.62 m/s, *P = .*917) (Figure 2B).

## Discussion

To our knowledge, this is the first study to evaluate the change in stricture stiffness in patients with CD after biologics treatment using SWE. Shear wave speed significantly decreased 1 year after treatment in the UST group but not in the IFX group and the bio-switch groups. In contrast, BWT significantly decreased in all groups.

In patients with CD, intestinal stricture is characterized by focal asymmetric, transmural, and granulomatous inflammation affecting any segment of the gastrointestinal tract and can be subdivided into the 3 different types: predominantly fibrotic, inflammatory, and mixed.^23^ It is difficult to determine the type of stricture based on clinical manifestations and using serological indicators. Strictures containing considerable fibrosis are thought to be associated with intramural SWS indices, which may be higher in the fibrotic strictures than in purely inflammatory strictures. Regarding bowel wall SWS, it may be possible to evaluate the presence of fibrosis at the time of initial diagnosis.^24^ Fibrosis results from the activation of mesenchymal cells by TNF-α, TGF-β, vascular growth factor, insulin-like growth factor-1, matrix metalloproteinases, and other mediators released by leukocytes, epithelial and mesenchymal cells, and the gut microbiota. Transforming growth factor-β induces myofibroblast transformation from fibroblast and epithelial cells, and myofibroblast produced collagen and fibronectin leading extracellular matrix and causing fibrotic strictures. Thus TGF-β is an important molecular target against fibrotic strictures. The healing process could be different depending on the biologics, because of the different roles of target molecules.

Ustekinumab may suppress the activity of TGF- β activated by IL-22 and IL-17 because UST blocks IL-23 and also decreases the expression of IL-17A downstream, which may lead to suppression of fibrogenesis and stenotic formation.^25^ Ustekinumab indirectly downregulates TNF-α expression, which slows the healing of ulcerations, as compared with anti-TNF-α agents. Thus, UST may lead to the suppression of fibrogenesis and the formation of stenosis. Murate et al^26^ reported that UST could be an effective treatment for preventing restenosis of the small bowel after endoscopic balloon dilation in 2 cases with small bowel lesions. A Finnish study showed that among 84 of 155 patients with a structuring phenotype, only 26 (17%) patients required surgery over the 2-year study period.^27^ Conversely, anti-TNF-α antibodies induced small bowel stenoses in 8 of 15 (53.3%) patients after 6-22 maintenance infusions.^28^ In another prospective study investigating the frequency of small bowel obstruction in CD stricture patients after IFX or adalimumab, 2 of 9 (22.2%) had bowel obstruction requiring surgical resection.^29^ However, data on the development of bowel obstruction after UST is lacking. Further prospective studies are required to increase the sample size and extend a number of the observation period after the biologics therapy.

Two studies evaluated intestinal lesions using SE in patients with CD treated with anti-TNF therapy. Orlando et al^30^ monitored the outcomes of anti-TNF therapy and revealed no statistically significant difference in strain ratio values at baseline and at 14 and 52 weeks after therapy. Our results also indicated no statistically significant difference in SWE indices in the IFX and the bio-switch groups. Only the SWS in the UST group was significantly decreased 1 year after treatment. The lack of improvement in the bio-switch group may be due to the fibrotic strictures formed during the long duration of CD. Our results suggested that early induction of UST can be related to the suppression of fibrosis; however, studies performing immunohistochemical analysis of stenosis lesion treated with UST are warranted to confirm our findings. 

Bowel wall thickness may be useful in monitoring biologics-induced bowel activity improvement in patients with CD.^10,22^ An increase in the echogenicity of the third layer of the intestinal wall (submucosal layer) is observed, which is currently thought to be an expression of submucosal fibrosis, whereas hypoechogenicity of the intestinal layers is related to hyperemia and edema. Increased BWT is an important component of inflammation,^8^ and improved lesions are generally defined as those with improvement >1 mm or normalization of BWT.^13^ The current review described patients with risk factors, as such wall thickness of less than 10 mm, who were referred for early surgery or dilatation. In this study, BWT in all groups, including the bio-switch group, significantly decreased after biologics treatments, and both biologics improved simple CDAI regardless of bio-naïve status. Additionally, BWT in all groups was <10 mm. Therefore, BWT may be a beneficial tool to evaluate activity mainly reflecting mainly inflammation, whereas SWS may be a better tool to evaluate fibrotic stricture reflecting tissue stiffness. However, there is a need to evaluate not only BWT but also other US factors, such as stricture length and prestenotic dilatation, in further study. 

This study had several important limitations. First, the sample size was small. Second, a reference was not established, such as histological fibrosis analysis using surgical resection organs. Third, this study was lack of randomization of treatment. Fourth, the baseline of clinical data of the three groups was not matched; therefore, selection bias should be considered. In particular, the baseline CDAI differed between the IFX and UST groups. Almost all patients with CD as well as perianal and perirectal lesions were treated with IFX because IFX is more effective for perianal lesions than UST. Thus, further prospective randomized studies evaluating the development of obstruction after biologics treatment are required to confirm the difference in SWS. In addition, a multicenter large-scale study adjusting the clinical data between the UST and IFX groups is required to confirm the results. 

## Conclusion

In conclusion, SWE may be a useful method for evaluating stiffness in areas of intestinal stricture in patients with CD. However, a prospective randomized study evaluating the development of obstruction after biologics treatment is warranted to validate our findings.

## Figures and Tables

**Table 1.** Clinical Background of Crohn’s Disease Patients receiving Biologic Treatments

		IFX n = 6	UST n = 8	IFX→UST n = 7	*P*
Age, mean ± SD		25.3 ± 13.7	37.6 ± 22.2	47.4 ± 10.5	.869^c^
Gender (male/female)		4/2	4/4	5/2	NS^b^
BMI: median (IQR)		17.0 (16.1-17.5)	19.8 (17.5-21.3)	21.2 (20.7-22.1)	.281^c^
Disease duration (years): median (IQR)		1.5 (1.0-1.6)	1.7 (1.0-2.0)	19.0 (18.0-29.0)	.003^c^
Bionaive case (%)		6 (100%)	8 (100%)	—	—
Duration of biologics (month): median (IQR)		17.0 (12.0-28.5)	22.0 (19.0-29.7)	228 (204-336)	.001^a^
Duration of UST (months): median (IQR)		—	22.0 (19.0-29.7)	21.0 (20.0-29.0)	.789^a^
History of operations (%)		0	1 (13%)	6 (86%)	.014^b^
Obstruction/ileus (%)		1 (16%)	3 (38%)	3 (43%)	NS^b^
Endoscopic balloon dilatation (%)		1 (16%)	1 (13%)	3 (43%)	NS^b^
Stricture location					
Ileum		2 (33%)	4 (50%)	7 (100%)	NS^b^
Bauhin’s valve		6 (100%)	4 (50%)	7 (100%)	
Endoscopically impassable stricture		6 (100%)	4 (50%)	7 (100%)	NS^b^
Intestinal narrowing (<3 mm) by x-ray exam		6 (100%)	4 (50%)	7 (100%)	
Clinical Data					
Simple CDAI	Baseline; median (IQR)	5.5 (2.0-11.0)	2.0 (0.5-2.75)	1.0 (1.0-3.0)	.076^a^
	1 year after; median (IQR)	0 (0-1.5)	0.5 (0-2.5)	0 (0-0)	.347^a^
	P^*^	.027^e^	.063^e^	.024^e^	
CRP	Baseline; median (IQR)	4.26 (2.22-13.7)	0.85 (0.29-5.19)	0.65 (0.6-1.65)	.058^a^
	1 year after; median (IQR)	0.03 (0.02-0.23)	0.07 (0.03-0.19)	0.63 (0.08-0.85)	.046^a^
	P^*^	.028^e^	.018^e^	.204^e^	
Hemoglobin	Baseline; mean ± SD	11.4 ± 2.3	11.9 ± 1.5	12.5 ± 1.8	.584^c^
	1 year after; mean ± SD	13.9 ± 0.8	13.5 ± 1.3	12.9 ± 0.8	.232^c^
	P^*^	0.021^d^	0.023^d^	0.465^d^	
Albumin	Baseline; mean ± SD	2.9 ± 0.5	3.7 ± 0.6	3.7 ± 0.7	.053^c^
	1 year after; mean ± SD	4.2 ± 0.2	4.1 ± 0.4	3.8 ± 0.2	.030^c^
	P^*^	.001^d^	.092^d^	.73^d^	

CRP, C-reactive protein; IFX, infliximab; IQR, interquartile range; IFX→UST, bio-switch from IFX to UST; NS, not significant; UST, ustekinumab.

*P*^a^ by Kruskal–Wallis test;* P*^b^ by chi-square test;* P*^c^ by 1-way ANOVA test; *P*^d^ paired *t*-test; *P*^e^ Wilcoxon test; P*, Comparison before and after treatment.

**Table 2.** Comparison of the Changes in Bowel Wall Thickness and Shear Wave Speed Analyzed by US in Crohn’s disease patients receiving Biologic Treatment

	IFX (n = 6)			UST (n = 8)			IFX→UST (n = 7)			P^*^		
	Baseline	1 year after	Subtraction: baseline from 1 year after	Baseline	1 year after	Subtraction: baseline from 1 year after	Baseline	1 year after	Subtraction: baseline from 1 year after	Baseline	1 year after	Subtraction
Bowel wall thickness of stricture (mm): median (IQR)	4.4 (3.85 to 7.5)	2.89 (2.19 to 3.69)	−1.51 (−4.61 to 1.07)	4.88 (4.15 to 6.48)	3.97 (3.78 to 4.17)	−1.03 (−2.66 to 0.39)	6.7 (6.02 to 7.48)	5.15 (4.02 to 6.17)	−1.2 (−2.0 to 0.53)	.176	.004	.413
Shear wave speed (Vs:m/s): median (IQR)	2.24 (1.99 to 2.63)	2.07 (1.88 to 2.39)	−0.22 (−0.12 to 0.99)	2.81 (2.20-4.48)	1.65 (1.53 to 1.76)	−1.58 (−3.28 to 0.67)	3.5 (2.95 to 4.04)	3.62 [2.77 to 4.39]	−0.28 (−1.74 to 0.89)	.034	.001	.031

IFX, infliximab; IFX→UST, bioswitch from IFX to UST; IQR, interquartile; UST, ustekinumab.
